# Herpes simplex virus-2 infections in pregnant women from South Africa: Evaluation of the ImmunoFLOW rapid test

**DOI:** 10.4102/ajlm.v9i1.854

**Published:** 2020-08-31

**Authors:** Shanthie Govender, Lungile Mbambo, Makandwe Nyirenda, Motshedisi Sebitloane, Nathlee Abbai

**Affiliations:** 1 School of Clinical Medicine Research Laboratory, Nelson Mandela School of Medicine, University of KwaZulu-Natal, Durban, South Africa; 2 South African Medical Research Council, HIV Prevention Research, Durban, South Africa; 3 Department of Obstetrics and Gynecology, School of Clinical Medicine Research Laboratory, Nelson Mandela School of Medicine, University of KwaZulu-Natal, Durban, South Africa

**Keywords:** HSV-2 infection, pregnant women, rapid test, South Africa

## Abstract

The diagnostic performance of ImmunoFLOW, a rapid test for detecting herpes simplex virus type-2 (HSV-2) infections, was investigated in 248 antenatal women. Approximately one hundred and seventy-seven (71%) of the enrolled women were infected with HSV-2. Sero-positivity was associated with older age ([≥ 30 years] 104/177, 58%), having a secondary level of education but not tertiary level of education (125/177, 70.6%), and being unmarried (150/177, 84.7%). The sensitivity of the ImmunoFLOW test in relation to the HerpeSelect HSV-2 enzyme-linked immunosorbent assay was 89.7% and specificity was 96.2%. The ImmunoFLOW therefore can serve as a valuable test in screening for HSV-2 infections in pregnant women.

## Introduction

The South African HIV/AIDS and Sexually Transmitted Infections (STI) Strategic Plan deems the prevention and early treatment of STIs to be a major health priority for the country; emphasis has been placed on the delivery of quality services for testing and treating of STIs.^1^ Simple, rapid and affordable point-of-care tests for diagnosing STIs may be advantageous, because patients can be tested and treatment commenced in a single visit.^2^ Cassette- and strip-based point-of-care tests are highly applicable for use in resource-poor settings, because they do not require electricity, a laboratory or highly trained staff to perform the testing.^3^ Additionally, such tests could have tremendous potential for use in community clinics in an STI-endemic country such as South Africa.

In highly endemic herpes simplex virus type-2 (HSV-2) regions such as South Africa,^4,5^ routine screening for HSV-2 infections would pose a huge financial burden on the health system, because the only available diagnostic tests for HSV-2 are enzyme-linked immunosorbent assays (ELISA), which are expensive and time consuming. Due to the high turn-around time to receive test results, many infected individuals may be lost to follow up. In addition, ELISA requires the use of specialised equipment and highly trained laboratory staff. To address these limitations, rapid point-of-care tests that: are easy to operate, provide results on the same day and are relatively inexpensive would result in a larger number of testing and subsequent treatment infected individuals. The ImmunoFLOW HSV Test (GenBio, San Diego, California, United States) is a point-of-care cassette test that detects IgG to HSV gG-2 (specific HSV type 2 and total HSV [type 1 + type 2]) for type-specific classification, which is not possible using whole virus lysate, and also provides epidemiological information on these diseases. Currently, the diagnostic performance of this test has not been evaluated in a South African setting. Additionally, there is a lack of published research studies on this point-of-care test, both locally and globally.

This study compared the results of the ImmunoFLOW with the HerpeSelect HSV-2 ELISA (Focus Diagnostics, Cypress, California, United States). We used the HerpeSelect HSV-2 ELISA from Focus Diagnostics as our reference test, because this test has been approved by the manufacturer and has a certification mark approval (GmbH, Hannover, Germany) for detection of HSV antibodies in pregnant women globally.

## Methods

### Ethical considerations

The study and all study related materials were approved by the Biomedical Research Ethics Committee, University of KwaZulu-Natal (BE392/17).

### Study setting and population

The study was conducted between April 2017 and August 2017 at the King Edward VIII Hospital antenatal clinic in Durban, KwaZulu-Natal, South Africa. Two hundred and forty-eight women participated in this study. During screening, an estimated 20% of the women approached refused study participation. The study criteria included: being pregnant and aged 18 years or older, willing to give written informed consent, willing to undergo a blood draw, and willing to allow the study team to document their HIV status from their clinic cards.

### Data and specimen collection

For this study, data on the women’s demographics and clinical information were recorded on a case report form. Women who had symptoms of genital ulcers and sores were treated by syndromic management. For the syndromic management approach, patients presenting with a genital ulcer or sore were treated with aciclovir, oral, 400 mg 8-hourly for 7 days.

Venous blood (3.5 mL) was collected by a hospital professional nurse. The blood was collected into a serum separator gel tube. The blood was processed and tested at University of KwaZulu-Natal, School of Clinical Medicine Research Laboratory.

### ImmunoFLOW test

The samples were tested according to the manufacturer’s instructions. Test cassettes were placed on a dry level surface, and 100 *µ*L of wash solution was added to the cassette. The patient samples were diluted in sample diluent and thereafter 200 *µ*L of the dilution was added to the cassette. A second wash step was conducted before the addition of 100 *µ*L of Color G to the cassette. A final wash step was performed and the results were available within 2 min. Each cassette included a reagent-positive control. The presence of a red or pink dot in the individual test and control windows was read as a positive result.

### HerpeSelect HSV-2 enzyme-linked immunosorbent assay

The HerpeSelect HSV-2 ELISA assay is a glycoprotein G-based type-specific ELISA technique which produces qualitative results. Two hundred ul of each serum sample was tested according to the manufacturer’s recommendations (Focus Diagnostics, Cypress California, United States). Controls that were provided with kits were included for all runs: IgG High Positive Control (index value greater than 3.5); IgG Low Positive Control (index value between 1.5–3.5); and Negative Control (index value less than 0.8). The cut-off value used to determine a positive result was > 1.10 index value; 0.9–1.10 index values were considered equivocal and index values < 0.9 were considered as negative results. All samples that produced equivocal index values were re-tested.

### Data analysis

All analyses were performed using STATA, version 14 (StataCorp LLC, College Station, Texas, United States). The diagnostic performance (i.e. sensitivity, specificity, positive predictive value, and negative predictive value) of the ImmunoFLOW test was compared to the gold standard HerpeSelect HSV-2 ELISA. A *p*-value of < 0.05 was considered as significant.

## Results

A prevalence of 177/248 (71.4%) for HSV-2 was observed on the ImmunoFLOW test and prevalence was 195/248 (78.6%) on the HerpeSelect HSV-2 ELISA. The prevalence of HIV in this population was 124/248 (50.0%). Approximately (107/177) of the women were positive for HSV-2 and were also HIV-positive. The majority of the women who tested positive for HSV-2 were older than age 30 years (104/177, 58%, *p* = 0.001), had completed secondary education but not tertiary education (125/177, 70.6%, *p* = 0.05), were unemployed (107/177, 60.5%, *p* = 0.55), were unmarried (150/177, 84.7%, *p* = 0.02) and reported having more than one lifetime sexual partners (164/177, 92.6%, *p* = 0.0002) (Table 1).

**TABLE 1:** Description of pregnant women recruited from the antenatal clinic of the King Edward VIII Hospital in Durban, KwaZulu-Natal, South Africa, 2017.

Variable	HSV-2 status by ImmunoFlow						*p*
	Total		Positive		Negative		
	*N*	%	*N = 177*	*% (n/N)*	*N = 71*	*% (n/N)*	
**Socio-demographic factors**							
**Age (years)**							
18–24	43	17.3	26	14.7	17	23.9	0.018
25–29	75	30.2	47	26.6	28	39.4	
30–34	60	24.2	48	27.1	12	16.9	
≥ 35	70	28.2	56	31.6	14	19.7	
**Level of education**							
Primary	12	4.8	9	5.1	3	4.2	0.11
Secondary	162	65.3	125	70.6	37	52.1	
Tertiary	74	29.8	43	24.3	31	43.7	
**Employed**							
Yes	101	40.7	70	39.5	31	43.7	0.551
No	147	59.3	107	60.5	40	56.3	
**Married**							
Yes	47	19.0	27	15.3	20	28.2	0.019
No	201	81.0	150	84.7	51	71.8	
**Behavioural factors**							
**Has regular partner**							
Yes	244	98.4	175	98.9	69	97.2	0.340
No	4	1.6	2	1.1	2	2.8	
**Living with current partner**							
Yes	107	43.1	79	44.6	28	39.4	0.516
No	137	55.2	96	54.2	41	57.7	
Refused to answer	4	1.6	2	1.1	2	2.8	
**Age at first sex (years)**†							
< 15	6	2.4	5	2.8	1	1.4	0.449
15–19	164	66.4	118	67.0	46	64.8	
20–24	65	26.3	47	26.7	18	25.4	
25–29	9	3.6	5	2.8	4	5.6	
≥ 30	3	1.2	1	0.6	2	2.8	
**Lifetime number of sex partners**							
1	30	12.1	13	7.3	17	23.9	0.001
2–4	154	62.1	115	65.0	39	54.9	
≥ 4	64	25.8	49	27.7	15	21.1	
**Partner has other partners**							
Yes	66	26.6	49	27.7	17	23.9	0.785
No	74	29.8	51	28.8	23	32.4	
Don’t know	108	43.5	77	43.5	31	43.7	
**Condom use during pregnancy**							
Never	92	37.1	57	32.2	35	49.3	0.011
Sometimes	117	47.2	86	48.6	31	43.7	
Always	39	15.7	34	19.2	5	7.0	
**Clinical/biological factors**							
**Trimester of pregnancy**							
First	56	22.6	39	22.0	17	23.9	0.671
Second	119	48.0	83	46.9	36	50.7	
Third	73	29.4	55	31.1	18	25.4	
**HIV status (at enrolment)**							
Negative	122	49.2	69	39.0	53	74.6	0.000
Positive	124	50.0	107	60.5	17	23.9	
Refused	2	0.8	1	0.6	1	1.4	
**Contraceptive used before pregnancy**							
None	85	34.3	57	32.2	28	39.4	0.704
Condom	39	15.7	31	17.5	8	11.3	
Oral	16	6.5	11	6.2	5	7.0	
Injection	96	38.7	69	39.0	27	38.0	
Implant	12	4.8	9	5.1	3	4.2	
**Current symptoms of genital ulcers or sores**							
Yes	158	63.7	118	66.7	40	56.3	0.126
No	90	36.3	59	33.3	31	43.7	
**Treated for STI**							
Yes	130	52.4	102	57.6	28	39.4	0.010
No	118	47.6	75	42.4	43	60.6	
**STI symptoms**							
None	136	54.8	89	50.3	47	66.2	0.087
Abnormal vaginal discharge or foul-smelling odour	27	10.9	19	10.7	8	11.3	
Genital itching, warts or sores	63	25.4	52	29.4	11	15.5	
Multiple	22	8.9	17	9.6	5	7.0	

STI, sexually transmitted infections; HSV-2, herpes simplex virus type-2.

† , The number of participants (*n*) for age at first sex is 247 due to missing data for one participant.

The sensitivity of the ImmunoFLOW test was 89.7% and its specificity was 96.2%. Of the 248 samples tested, 175 samples were correctly classified as positive by the ImmunoFLOW test. However, there were 2 samples that the ImmunoFLOW test classified as positive whereas the reference test classified these as negative. In addition, 20 samples were falsely classified as negative by the ImmunoFLOW (Table 2). The positive predictive value of the ImmunoFLOW was 98.9% and its negative predictive value was 71.8%. The overall predicitive accuracy of the ImmunoFLOW test was 91.1% (95% confidence interval: 86.9% – 94.4%) (Table 3).

**TABLE 2:** ImmunoFLOW rapid test results compared to HerpeSelect 2 enzyme-linked immunosorbent assay results in antenatal women, KwaZulu-Natal, South Africa, 2017.

Reported symptoms and other infections	ImmunoFlow	HerpeSelect 2 ELISA	
		Positive	Negative
All women in the sample (***n*** = 248)	Positive	175	2
	Negative	20	51
No symptoms of genital ulcers or sores (***n*** = 90)	Positive	58	1
	Negative	9	22
Symptoms of genital ulcers or sores (***n*** = 158)	Positive	117	1
	Negative	11	29
HIV-negative (***n*** = 122)	Positive	67	2
	Negative	9	44
HIV-positive (***n*** = 124)	Positive	107	0
	Negative	10	7
HIV-negative and no symptoms of genital ulcers or sores (***n*** = 49)	Positive	25	1
	Negative	4	19
HIV-negative and symptoms of genital ulcers or sores symptoms (***n*** = 73)	Positive	42	1
	Negative	5	25
HIV-positive and no symptoms of genital ulcers or sores (*n* = 39)	Positive	32	0
	Negative	4	3
HIV-positive and reported no symptoms of genital ulcers or sores (*n* = 85)	Positive	4	0
	Negative	6	75

ELISA, enzyme-linked immunosorbent assay.

**TABLE 3:** Performance of the ImmunoFLOW rapid test compared to the HerpeSelect 2 enzyme-linked immunosorbent assay in antenatal women, KwaZulu-Natal, South Africa, 2017.

Statistic	All women in the sample			Reported symptoms of genital ulcers/sores			HIV-positive			HIV-negative and reported no genital ulcers/sores symptoms		
	Value (%)	95% CI	*n*	Value (%)	95% CI	*n*	Value (%)	95% CI	*n*	Value (%)	95% CI	*n*
Sensitivity	89.7	84.60–93.62	-	91.4	85.14–95.63	-	91.5	84.84–95.83		86.2	68.34–96.11	-
Specificity	96.2	87.02–99.54	-	96.7	82.78–99.92	-	100.0	59.04–100.0	-	95.0	75.13–99.87	-
Positive predictive value	98.9	95.74–99.71	-	99.2	94.45–99.88	-	100.0	-	-	96.2	78.63–99.41	-
Negative predictive value	71.8	62.66–79.49	-	72.5	59.88–82.32	-	41.2	27.90–55.87	-	82.6	65.54–92.23	-
Correctly classified	91.1	86.88–94.36	-	92.4	87.11–96.01	-	91.9	85.67–96.06	-	89.8	77.77–96.60	-
Number	-	-	-	-	-	158	-	-	124	-	-	49

*N* = 248.

CI, confidence interval.

## Discussion

The 78.6% prevalence of HSV-2 reported in this study was found to be higher than other published studies on antenatal women.^6,7,8,9,10^ A high prevalence of HSV-2 was observed in women who had more partners, thereby emphasising the association between increased number of sex partners and risk of contracting STIs. The performance of the ImmunoFLOW was comparable to other published reports on HSV-2 rapid tests, which reported sensitivities and specificities > 90%.^11,12,13,14,15^ Other published studies on HSV-2 rapid tests have not reported on the performance of those tests in the presence of other viral infections, such as HIV, or genital symptoms relating to infection. In this study, the ImmunoFLOW rapid test performance was not shown to be negatively affected by HIV infection. The test yielded a sensitivity of 91.5% and specificity of 100% among HIV-positive women. In addition, the test was able to detect infection in women who were presenting with symptoms of genital ulcers or sores. Among women who were symptomatic, the sensitivity of the test was 91% and its specificity was 97%. This study highlights that having a test such as the ImmunoFLOW test could greatly contribute to early detection and treatment of women with herpes simplex viruses for improved outcomes for pregnant woman and their babies.

### Limitations

The limitations of the study were as follows: Western blotting could not be performed as a second confirmatory test due to the high cost of the tests. The testing was performed at a research laboratory by medical technicians and is not a reflection of how the test would be performed at a clinic. Medical technicians are more experienced at laboratory procedures and quality checks. If the test had been conducted by a clinic nurse who has no laboratory experience, the results may have been different. This is yet to be confirmed. However, conducting evaluation studies at antenatal clinics will be a future research consideration. The test required the use of serum. With slight modifications, such as using blood collected by finger-prick instead of serum, this test could serve as a valuable test in screening for HSV-2 infections. However, this needs to be evaluated.

### Conclusion

Overall, we have shown that the ImmunoFLOW rapid test performed well in relation to the ELISA. Rapid laboratory tests for diagnosis of STIs will directly contribute to United Nations Sustainable Development Goals that focus on improvement in health.^16^

## References

[1] Lewis DA, Maruma E Revision of the national guideline for first-line comprehensive management and control of sexually transmitted infections: What’s new and why? South Afr J Epidemiol Infect. 2009;24(2):6–9. 10.1080/10158782.2009.11441341

[2] Hsieh Y-H, Hogan MT, Barnes M, et al Perceptions of an ideal point-of-care test for sexually transmitted infections–A qualitative study of focus group discussions with medical providers. PLoS One. 2010;5(11):e14144 10.1371/journal.pone.0014144 21152386 PMC2994750

[3] Tucker JD, Bien CH, Peeling RW Point-of-care testing for sexually transmitted infections: Recent advances and implications for disease control. Curr Opin Infect Dis. 2013;26(1):73 10.1097/QCO.0b013e32835c21b0 23242343 PMC3635142

[4] Abbai NS, Wand H, Ramjee G Socio-demographic and behavioural characteristics associated with HSV-2 sero-prevalence in high risk women in KwaZulu-Natal. BMC Rese Note. 2015;8(1):1 10.1186/s13104-015-1093-0 PMC4423103 25940115

[5] Kenyon C, Colebunders R, Buve A, Hens N Partner-concurrency associated with herpes simplex virus 2 infection in young South Africans. Int J STD AIDS. 2013;24(10):804–812. 10.1177/0956462413482810 23970590

[6] Lima L, Padalecki G, Castro C, Cordeiro J, De Paula V Seroprevalence of human herpesvirus type 2 in a reference center for pregnant women in Rio de Janeiro, Brazil. VRR. 2017;22:20–21. 10.17525/vrrjournal.v22i1.327

[7] Domercant JW, Louis FJ, Hulland E, et al Seroprevalence of Herpes Simplex Virus type-2 (HSV-2) among pregnant women who participated in a national HIV surveillance activity in Haiti. BMC Infect Dis. 2017;17(1):577 10.1186/s12879-017-2674-4 28821230 PMC5563013

[8] Anjulo AA, Abebe T, Hailemichael F, Mihret A Seroprevalence and risk factors of herpes simplex virus-2 among pregnant women attending antenatal care at health facilities in Wolaita zone, Ethiopia. Virol J. 2016;13(1):43 10.1186/s12985-016-0501-y 26979484 PMC4793703

[9] Nakubulwa S, Kaye DK, Bwanga F, Tumwesigye NM, Nakku-Joloba E, Mirembe FM Incidence and risk factors for herpes simplex virus type 2 seroconversion among pregnant women in Uganda: A prospective study. J Infect Dev Countr. 2016;10(10):1108–1115. 10.3855/jidc.6874 27801374

[10] Perti T, Nyati M, Gray G, et al Frequent genital HSV-2 shedding among women during labor in Soweto, South Africa. Infect Dis Obstet Gynecol. 2014;2014:Article ID 258291:8 pages. 10.1155/2014/258291 PMC4054931 24963269

[11] Ashley RL, Eagleton M, Pfeiffer N Ability of a rapid serology test to detect seroconversion to herpes simplex virus type 2 glycoprotein G soon after infection. JCM. 1999;37(5):1632–1633. 10.1128/JCM.37.5.1632-1633.1999 PMC84860 10203544

[12] Wald A, Ashley-Morrow R Serological testing for Herpes Simplex Virus (HSV)–1 and HSV-2 infection. Clin Infect Dis. 2002;35(2):S173–S182. 10.1086/342104 12353203

[13] Philip SS, Ahrens K, Shayevich C, et al Evaluation of a new point-of-care serologic assay for herpes simplex virus type 2 infection. Clin Infect Dis. 2008;47(10):e79–e82. 10.1086/592696 18840082

[14] Laderman EI, Whitworth E, Dumaual E, et al Rapid, sensitive, and specific lateral-flow immunochromatographic point-of-care device for detection of herpes simplex virus type 2-specific immunoglobulin G antibodies in serum and whole blood. CVI. 2008;15(1):159–163. 10.1128/CVI.00218-07 18003814 PMC2223856

[15] Shevlin E, Morrow RA Comparative performance of the Uni-Gold™ HSV-2 Rapid: A point-of-care HSV-2 diagnostic test in unselected sera from a reference laboratory. J Clin Virol. 2014;61(3):378–381. 10.1016/j.jcv.2014.08.012 25200648 PMC4252648

[16] United Nations General Assembly Sustainable devlopment goals [homepage on the Internet]. 2015 [cited 2018 Jun 27]. Available from: https://www.un.org/sustainabledevelopment/sustainable-development-goals/

